# Assessing the Need for Adjuvant Chemotherapy After Stereotactic Body Radiation Therapy in Early-stage Non-small Cell Lung Carcinoma

**DOI:** 10.7759/cureus.901

**Published:** 2016-11-29

**Authors:** Raphaël Jumeau, Houda Bahig, Édith Filion, Marie-Pierre Campeau, Louise Lambert, David Roberge, Andrei-Bogdan Gorgos, Toni Vu

**Affiliations:** 1 Department of Radiation Oncology, Centre hospitalier de l'université de Montréal (CHUM); 2 Radiology, Centre hospitalier de l'université de Montréal (CHUM)

**Keywords:** lung cancer, sbrt, adjuvant chemotherapy

## Abstract

**Purpose:**

Surgery remains the standard treatment for medically operable patients with early-stage non-small cell lung carcinoma (NSCLC). Following surgical resection, adjuvant chemotherapy is recommended for large tumors >4 cm. For unfit patients, stereotactic body radiation therapy (SBRT) has emerged as an excellent alternative to surgery. This study aims to assess patterns of recurrence and discuss the role of chemotherapy after SBRT for NSCLC.

**Methods:**

We reviewed patients treated with SBRT for primary early-stage NSCLC between 2009 and 2015. Total target doses were between 50 and 60 Gy administered in three to eight fractions. All patients had a staging fluorodeoxyglucose (FDG) positron emission tomography (PET) integrated with computed tomography (CT) scan, and histologic confirmation was obtained whenever possible. Mediastinal staging was performed if lymph node involvement was suspected on CT or PET/CT. Survival outcomes were estimated using the Kaplan-Meier method.

**Results:**

Among the 559 early-stage NSCLC patients treated with SBRT, 121 patients were stage T2N0. The one-year and three-year overall survival rates were 88% and 70%, respectively, for patients with T2 disease, compared to 95% and 81%, respectively, for the T1 patients (p<0.05). The one-year and three-year local control rates were equal in both groups (98% and 91%, respectively). In T2 patients, 25 (21%) presented a relapse, among which 21 (84%) were nodal or distant. The median survival of T2N0 patients following a relapse was 11 months.

**Conclusion:**

Lung SBRT provides high local control rates, even for larger tumors. When patients relapse, the majority of them do so at regional or distant sites. These results raise the question as to whether adjuvant treatment should be considered following SBRT for larger tumors.

## Introduction

Lung cancer is the most common cause of death from cancer worldwide [1]. Among all histological types, NSCLC is the most frequent [2]. Although SBRT is being prospectively compared to surgical resection for medically operable patients, the standard management of early-stage NSCLC remains surgical lobectomy [3].

The role of adjuvant treatment after surgery has been studied extensively. In 1995 the NSCLC collaborative group [4] published the first meta-analysis supporting the use of adjuvant chemotherapy. More recently, a larger meta-analysis [5] based on 4,584 patients suggested that adjuvant cisplatin-based chemotherapy significantly improves survival. Current guidelines recommend adding chemotherapy after complete resection for patients with high-risk tumors: vascular invasion, wedge resection, visceral pleural involvement, unknown lymph node status and tumors >4 cm [6].

For patients who are medically unfit or who decline surgery, SBRT has emerged as the favored alternative. It provides local control rates comparable to surgery with low toxicity [7]. However, adjuvant treatment is rarely considered after lung SBRT, even for those with larger tumors. In this study, we present our results for patients treated with SBRT for high-risk early-stage NSCLC and discuss the potential benefit of adjuvant treatment.

## Materials and methods

### Patients and tumors

We retrospectively reviewed patients treated with SBRT for NSCLC at our institution between July 2009 and August 2015. Pretreatment workup included a diagnostic CT, PET/CT, bronchoscopy and pulmonary function testing with measurements of forced expiratory volume in one second (FEV1) and diffusing capacity of the lung for carbon monoxide (DLCO) [8].

Mediastinal staging (MS) was performed if lymph node involvement was suspected on CT or PET/CT. Histological confirmation was sought by bronchoscopy or transthoracic needle biopsy. When appropriate, gold fiducials were placed during percutaneous lung biopsy to allow tumor tracking. If the biopsy was impossible or inconclusive, radiological and clinical criteria were followed [9]. Measurements of the lesions were based on the largest dimension in axial view on the diagnostic CT. We defined central lesions as tumors within 2 cm of the proximal tracheobronchial tree or within 2 cm of other mediastinal structures [10].

### Treatment planning and delivery

SBRT was delivered using a variety of radiotherapy platforms: helical tomotherapy, CyberKnife® robotic radiotherapy (Accuray Inc., Sunnyvale, CA, USA) or isocentric linear accelerators with volumetric modulated arc therapy (VMAT). Dose schedules were 60 Gy in three to five fractions for peripheral lesions, and 50 Gy in five fractions or 60 Gy in eight fractions for central lesions.

Patients were treated either with near-real-time tumor tracking with CyberKnife® or using an internal target volume (ITV) with VMAT or helical tomotherapy. CyberKnife® tumor tracking was achieved using fiducials or, when tumor was sufficiently large and dense, using a soft tissue tracking technique (Xsight Lung, Accuray Inc., Sunnyvale, CA, USA) [11].

All patients had a noncontrast 4D planning CT scan in supine position. The gross tumor volume (GTV) was delineated on the expiratory phase of 4D CT and corresponded to the macroscopic tumor on pulmonary CT windows. For patients treated with VMAT or helical tomotherapy, a BodyFIX (Elekta, Stockholm, Sweden) whole body vaccum immobilization device was also used. ITV was based on tumor motion in extreme phases of the respiratory cycle. An additional planning tumor volume (PTV) margin of 5 mm was added to the GTV in fiducials or Xsight Lung cases, or to the ITV, alternatively.

### Follow-up and endpoints

After completion of SBRT, patients were first seen at three months for a detailed history and physical exam, then every six months thereafter. A chest CT scan was performed before each visit.

The primary endpoint was the local control (LC) evaluated by radiologists based on follow-up CT scans. LC was defined radiologically by the absence of a growing lesion within the involved lobe on sequential follow-up CT scans [12]. If lymph node involvement or metastatic evolution was suspected during follow-up, patients underwent whole-body PET/CT to confirm the non-local (nodal or distant) relapse. A histological confirmation of the relapse was performed when possible.

### Statistics

Statistical analysis was performed by SPSS software (SPSS Inc., Chicago, IL, USA). The local control, nodal control, distant control and survival outcomes were estimated using the Kaplan-Meier method. Data were collected and analyzed using Student's t-test, Chi square and Fisher's exact test. P values less than 0.05 were considered statistically significant.

IRB approval, number 09.029, was provided by Comité d'éthique de la recherche du CHUM. Patient informed consent was obtained.

## Results

### Population and tumor characteristics

Between July 2009 and August 2015, 574 treatments had been delivered in 559 patients. Patient and lesion characteristics are summarized in Table 1.

**Table 1:** Patients and tumors characteristics KPS: Karnofsky Performance Scale; FEV1: forced expiratory volume in one second; DLCO: diffusing capacity of the lung for carbon monoxide

	T1	T2	P value
Patients	438	121	
Age (years)	73	76	< 0.05
KPS	90	80	> 0.1
FEV1 (L)	1.2	1.3	> 0.1
FEV1 (%)	62	66	> 0.1
DLCO (%)	57	57	> 0.1
Lesions	451	123	
Size (cc)	1.8	3.6	< 0.05
Central/peripheral	77/374	52/71	< 0.05
Histology (%)	71	86	< 0.05
Adenocarcinoma	36	39	
Squamous cell carcinoma	20	31	
Large cell carcinoma	2	2	
Other specified carcinoma	6	9	
Mediastinal staging (%)	14	21	< 0.05

### Cohort outcomes

**Figure 1:**
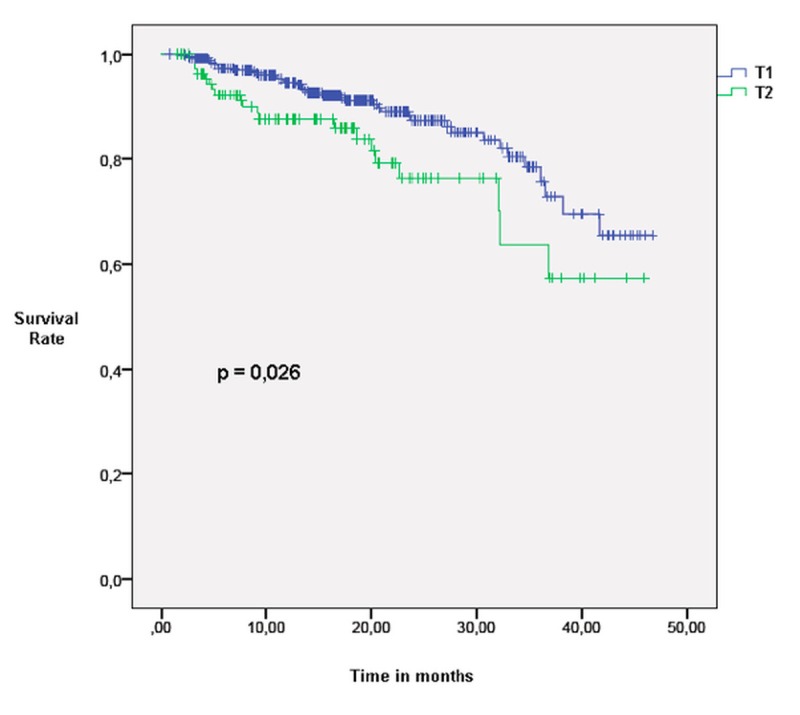
Overall survival in T1 and T2 patients

**Figure 2:**
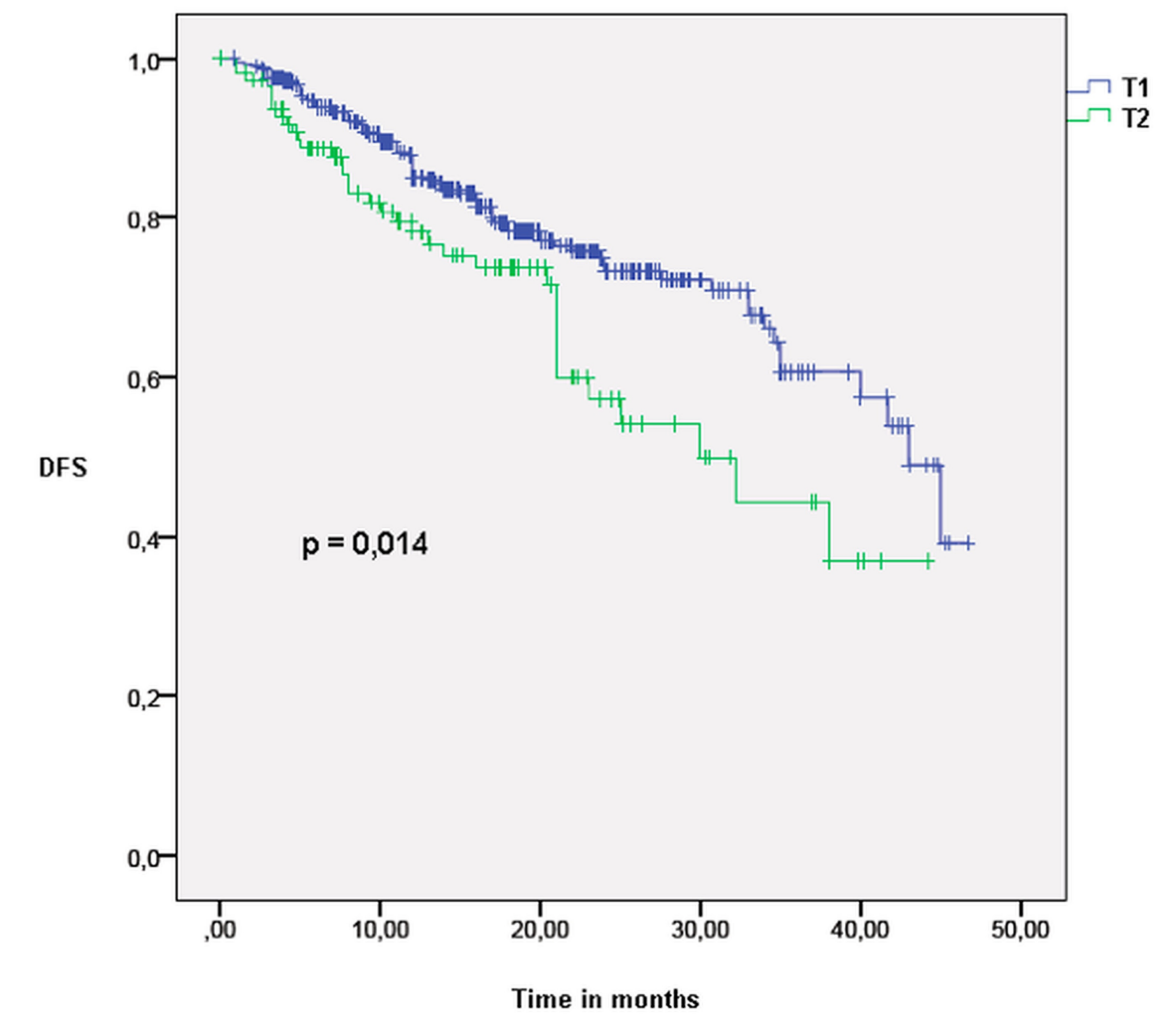
Disease-free survival in T1 and T2 patients

The median follow-up time was 16 months (range: one to 62). LC rates were equal in the T1 and T2 groups: 98% and 91% at one and three years, respectively. Non-local control rates were different between T1 and T2 patients: 93% vs. 89% at one year and 83% vs. 69% at three years (p<0.05), respectively. Overall survival (OS) at one and three years was also statistically different between the two groups: 95% and 81% in T1 patients and 88% and 70% in T2 patients (p<0.05), respectively (Figure 1). The percentage of deaths attributable to lung cancer was 42% in the T1 group, while it was 52% in the T2 group (p>0.1) (Table 2). Disease-free survival (DFS) was 88% at one year and 66% at three years in the T1 group, and it was 79% and 51% in the T2 group (p<0.05), respectively (Figure 2).

**Table 2:** Death statistics RR: relative risk CI: confidence interval

	T1	T2	P value
Total number of deaths	43	21	
Number of lung cancer deaths	18	11	
%	42	52	> 0.1
RR	1.25		
95% CI	[0.5 ; 4.3]		

### T2 patients

**Figure 3:**
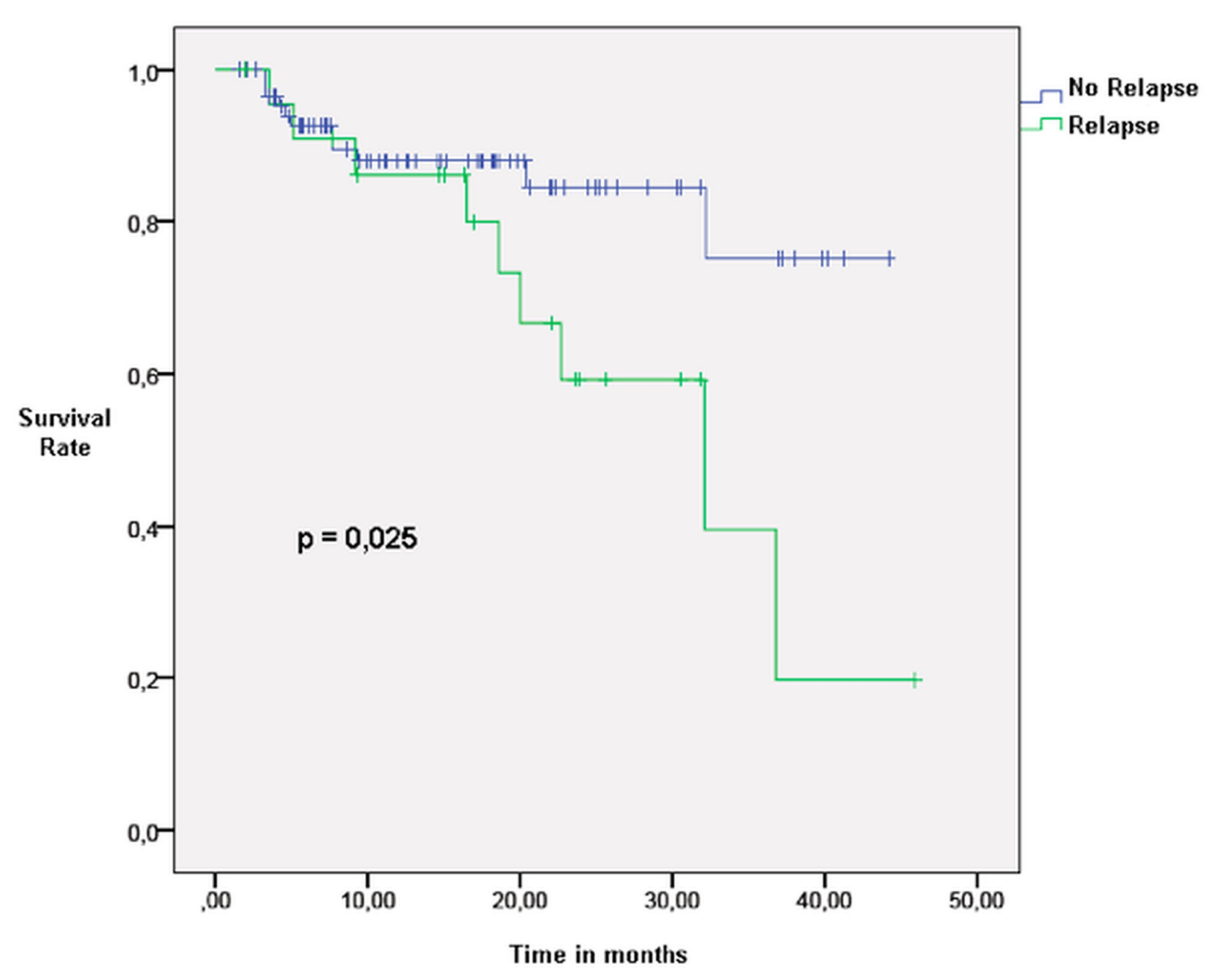
Overall survival in T2 patients after the diagnosis of relapse

Among the 121 T2 patients, six were staged T2b. Unsurprisingly, survival of T2 patients without relapse was higher than T2 patients having suffered a relapse (p<0.05) (Figure 3). The median survival of T2N0 patients following a relapse was 11 months. Twenty-five patients (21%) presented a relapse: four (16%) were local and 21 (84%) were nodal or distant. Among the non-local relapses, eight (38%) were nodal and 13 (62%) were distant. Two patients with distant relapse limited to the brain had local treatment (one radiosurgery and one surgery). In the rest of the non-local relapse group, six patients (32%) were considered eligible to receive palliative chemotherapy. One patient (5%) declined chemotherapy and three (16%) received cytotoxics, while two (10%) had targeted therapy.

## Discussion

Adjuvant chemotherapy is a well-established treatment after complete surgical resection of NSCLC tumors >4 cm. For nonsurgical patients, SBRT is now considered standard treatment. Even if SBRT is a highly efficient local treatment, there is a lack of data available concerning the possibility of adjuvant treatment after lung SBRT for large NSCLC tumors. In our study, we confirmed that SBRT provides excellent LC with a three-year rate of 91%. This result is comparable to rates previously reported for NSCLC SBRT [13-19]. Furthermore, LC is similar between the T1 and T2 groups and comparable to that reported in surgical series. In fact, Kastelijn et al. [20], in a retrospective study, compared clinical outcomes of SBRT (N= 53) versus surgical resection (N= 175) for stage I-II NSCLC. They showed similar results in terms of OS and progression-free survival between surgery and SBRT. Chang et al. [21], in a pooled analysis of two randomized trials comparing surgery and SBRT for operable stage I (T1a, T1b and T2a) NSCLC, found similar results between the two treatments in terms of LC and DFS; the three-year OS was higher for SBRT patients (95% vs. 79%, p=0.037), but the number of patients was not sufficient to conclude (N= 58). In our study, non-local recurrence rates were also comparable to surgical series. Robinson et al. [22] studied patterns of failure after surgery (lobectomy or pneumonectomy) or SBRT for stage I NSCLC. In their retrospective study, they concluded to an advantage in OS for surgery, but the primary tumor LC rate (98.7% vs. 95.3%, p=0.088), the regional control rate (82.9% vs. 78.1%, p=0.912) and the distant control rate (76% vs. 54%, p=0.152) were respectively similar for surgery versus SBRT. Moreover, in our study, we showed a clear majority of non-local recurrences for T2 patients. Comparable LC rates of lung SBRT with those of surgery, as well as the important rates of non-local recurrences, raise the question as to the benefit of an adjuvant treatment.

In a systematic review of the literature, Burdett et al. [23] showed that there is a clear advantage to add chemotherapy after surgery. Their analyses based on 8,447 patients over 34 trials proved an absolute increase of 4% in survival at five years. It must be pointed out that patients in these studies were younger (median age of 60 years) than lung cancer patients seen in clinical routine, including our practice. In an analysis of randomized trials by Madroszyk-Flandrin et al. [24], based on 71 patients with stage I-IV NSCLC, they showed that chemotherapy is feasible in elderly patients (≥70 years). In fact, elderly patients could receive adjuvant chemotherapy but had more modest survival outcomes than younger patients. As analyzed by Früh et al. [25], based on patient data from the Lung Adjuvant Cisplatin Analysis (LACE), elderly patients had worse outcomes. They reported a higher rate of noncancerous-related death, and elderly patients received lower doses of chemotherapy. Adding chemotherapy after SBRT seems possible, even for older patients, but a rigorous screening is necessary to select patients fit enough to receive the treatment. In recent years, targeted therapies have emerged in NSCLC management. These treatments are considered safer because patients have less side effects. However, the role of targeted therapies after surgery is not clear [26]. The most represented targeted agents are the tyrosine kinase inhibitors (TKI) of epidermal growth factor receptor (EGFR). The BR-19 trial [27] enrolled 503 patients in a phase III study comparing surgery alone and surgery followed by EGFR-TKI (gefitinib) administration, regardless of the EGFR status. There was no difference in OS or DFS between the two groups. More recently, the RADIANT trial [28] studied adjuvant EGFR-TKI (erlotinib) treatment after resection of stage IB-IIIA NSCLC only for tumors with the expression of EGFR. In the subgroup of patients with EGFR mutation, DFS favored erlotinib (46.4 vs. 28.5 months), but this was not statistically significant. Regarding these results, targeted agents may provide a benefit as adjuvant treatment and have the advantage to be better tolerated than cytostatic drugs. It must be pointed out that they seem not to be indicated for patients without specific mutations.

One retrospective study of 65 patients by Chen et al. [29] suggested a benefit for adjuvant chemotherapy in T1-3N0 NSCLC following SBRT. The three- and five-year OS rates in the adjuvant chemotherapy group were 81% and 46%, respectively, while they were 50% and 32%, respectively, for patients who had not received chemotherapy. Patients who received adjuvant chemotherapy had a lower relapse rate. But the number of patients who received adjuvant chemotherapy (N=17) was not sufficient to provide significant results.

In our study, only one third of T2 patients were eligible for systemic treatment after the diagnosis of relapse. The majority of these patients (68%) were unfit to receive systemic treatment and were directed to supportive care units. Although this may be an argument in favor of adjuvant treatment (when the patient may be more fit), it also highlights that a number of these patients may not be fit for chemotherapy after SBRT. OS and DFS are significantly worst in T2 patients. The shortness of the median survival time after diagnosis of relapse in the T2 group highlights the aggressiveness of the recurrence that is congruent with the median survival of 10-11 months described by Schiller et al. [30] in metastatic NSCLC receiving chemotherapy.

Adjuvant treatment for early-stage NSCLC after lung SBRT could potentially benefit selected patients after lung SBRT. However, SBRT patients tend to be older and frailer than patients eligible for surgery; therefore, fewer of them would be eligible for an adjuvant treatment. On the other hand, few patients received SBRT because they refused surgery, and they will probably refuse adjuvant treatment even if they are eligible for it. Nonetheless, our results showed that patients with larger tumors have a worse outcome, so further prospective studies with bigger cohorts are warranted.

## Conclusions

Lung SBRT provides excellent local control rates, even for larger tumors. Overall survival and patterns of failure are similar to surgery. However, it remains unclear whether SBRT patients will be sufficiently fit for adjuvant chemotherapy, but these results show the need for future trials to study this question.
